# Gas Chromatography Method for Quantitation of Residual Solvent Impurities in Nanoformulations

**DOI:** 10.3390/mps9040110

**Published:** 2026-07-23

**Authors:** Krishna Kattel, Rachael M. Crist, Jeffrey D. Clogston

**Affiliations:** Nanotechnology Characterization Laboratory, Cancer Research Technology Program, Frederick National Laboratory for Cancer Research, Frederick, MD 21701, USA; krishna.kattel@nih.gov (K.K.); rachael.crist@nih.gov (R.M.C.)

**Keywords:** volatile organic impurities, flame ionization detector, nanomedicine, residual solvents, liposome, polymeric nanoparticles, headspace gas chromatography, short chain fatty acids, formic acid

## Abstract

The development and validation of a sensitive, rapid, and specific gas chromatography method for the evaluation of 19 common Class 2 and Class 3 solvents frequently used in nanomedicine formulation is described. Method validation was performed using PerkinElmer’s headspace gas chromatograph system with flame ionization detection and an Elite 624 Crossbond 6% cyanopropylphenyl-94% dimethylpolysiloxane or DB-Fatwax-Ultra Inert column with helium as the carrier gas. Validation characteristics such as linearity, spike recovery, method precision, specificity, sensitivity, limit of detection/quantitation, and analyte stability were evaluated. The validated methods showed excellent linearity, with a correlation coefficient > 0.99, and good precision, with intra-day precision < 7.4% for all tested analytes. The percent recoveries ranged 83–104% within the method’s quantitation range. In comparison to previously reported methods, the current method has a much shorter equilibration time, higher sensitivity, better separation for many solvents, and a wide concentration detection range. The current method is also perfectly suitable to analyze short chain fatty acids such as formic acid, acetic acid, butyric acid, and valeric acid without requiring additional extraction or derivatization steps. Notably, the method was found to be suitable for analysis of formic acid—a common solvent in certain nanoformulations and one in which there is no prior gas chromatography method available which does not require this additional sample manipulation—down to approximately 75 ppm. Herein, the method is demonstrated using various nanoformulations, including the commercial Doxil formulation as well as several research nanoformulations, including polymeric, cross-linked polymeric, and dendrimer platforms.

## 1. Introduction

Various organic solvents are used in the synthesis of complex drug products such as nanomedicines, in the manufacturing and purification of products that may be used as starting materials or excipients, and for cleaning and maintenance of equipment used in manufacturing processes. Volatile short chain fatty acids (SCFAs) also have a wide range of pharmaceutical applications. For example, formic acid is used for chemical synthesis and pharmaceutical processing, acetic acid is used for improving the solubility of weakly basic active pharmaceutical ingredients (APIs), and butyric acid is used for production of disinfectants [1,2,3]. These residual solvents (RS) are considered drug product impurities, offering no therapeutic benefit. Irregular and unregulated concentrations of these residual volatile impurities not only pose health risks to patients but also affect the product’s quality [4,5,6,7]. In addition, RS can affect the physicochemical properties of nanotherapeutics, such as particle size, dissolution, wettability, and may affect the loading and release of drug [8]. Therefore, detection and quantitation of residual volatile impurities is mandatory for quality control to ensure drug products meet the compendial requirements, safeguarding patients’ welfare.

The level of RS tolerated in final drug products is well-described in pharmacopeias such as the United States Pharmacopeia (USP) [9] and European Pharmacopeia (EP) [10] and is closely monitored by regulatory agencies, including the United States Food and Drug Administration (US FDA), United Kingdom’s Medicines and Healthcare products Regulatory Agency (MHRA), and Australia’s Therapeutic Goods Administration (TGA) among others. The International Council for Harmonization of Technical Requirements for Registration of Pharmaceuticals for Human Use (ICH) and FDA have released a guideline for classification of residual solvents—Impurities: Guidelines for Residual Solvents Q3C(R9) [11,12]. Per this guidance, RS were evaluated for their possible risk to human health and placed into one of three classes based on their toxicity data and environmental impact. Class 1 solvents such as benzene, carbon tetrachloride, 1,2-dichloroethane, 1,1-dichloroethene, and 1,1,1-trichloroethane are known to have unacceptable toxicities and deleterious environmental effects; the ICH limit for these solvents is <10 ppm. Class 1 solvents should be completely avoided unless they are absolutely required in the production of drug products that provide significant therapeutic advances; thus, their analysis is beyond the scope of this study. Class 2 solvents include acetonitrile, chlorobenzene, chloroform, cyclohexane, hexane, methanol, N-methyl-2-pyrrolidone, N,N-dimethylformamide (N,N-DMF), tetrahydrofuran (THF), xylene, toluene, pyridine, ethylene glycol, dichloromethane, 1,4-dioxane, and others. These solvents are non-genotoxic carcinogens and can induce neurotoxicity and, as such, their usage should be limited in drug products. Each Class 2 solvent has its own individual limit, with ICH limits ranging 50–3880 ppm based on the solvents’ permitted daily exposure (PDE). Class 3 solvents such as dimethyl sulfoxide (DMSO), acetone, ethanol, ethyl acetate, formic acid, heptane, propanol, 1-butanol, anisole, butyl acetate, diethyl ether, ethyl formate, acetic acid, and others are more innocuous compared to the Class 2 solvents, with lower toxic potential. Class 3 solvents may be used in drug products without justification and are typically limited to 5000 ppm or 0.5% (*w*/*w*).

Over the years, various methods have been reported for the quantitation of RS, including loss of weight upon drying, infrared spectroscopy, nuclear magnetic resonance spectroscopy, thermogravimetric analysis, differential scanning calorimetry, and most commonly, gas chromatography (GC) [8,13,14,15,16,17,18]. GC has become the preferred analytical technique as others suffer from low sensitivity, poor specificity, and high sample requirements. Several GC approaches are employed in the detection and quantification of RS, including direct (liquid) injection and headspace gas chromatography (HS-GC). Further, two types of HS auto-sampling techniques have been developed—dynamic HS and static HS [19]. Dynamic HS has higher sensitivity as compared to static HS; however, it is not as prone to automation like static HS [14,20]. Although HS sampling cannot match the sensitivity of direct injection, particularly for solvents with low volatility such as N,N-DMF, DMSO, etc., direct injection decreases column longevity and precision of the results due to carryover of analytes [13]. By using HS, only volatile components are introduced into the GC system, resulting in an extended column lifetime and reduced instrument maintenance.

At present, HS-GC with flame ionization detection (FID) is one of the most common analytical tools for the analysis of RS in API, excipients, and drug products [21,22,23,24,25,26,27,28,29]. A previously described GC-FID method from Bernardoni et al. showed the analysis of 30 RS in pharmaceuticals using hydrogen as the carrier gas and suggested replacement of helium with hydrogen as a more sustainable choice and recommended updating USP and EP compendia to state such [22]. Kay et al. reported a HS-GC method for quantitation of 25 common RS in pharmaceuticals, including water, using thermal conductivity detectors, showing the versatility of the method to analyze both water and RS simultaneously [30]. Methods for simultaneous detection of various solvents have been previously described for many pharmaceutical products and precursors, including for semi-volatile solvents (acetic acid, DMSO, and ethylene glycol), triethylamine and DMSO, low and high boiling RS (acetone, dichloromethane, n-butyl ether, and DMSO), and high boiling point volatile compounds in low boiling matrices (e.g., N,N-DMF, DMSO and others in water) [31,32,33,34]. While these methods are reliable for examining the specified few RS, they are either unable to provide broad analysis of other low volatile RS, including formic acid and other SCFAs, or lack overall sensitivity.

The analysis of SCFAs has been performed by combining various extraction methods—including liquid–liquid extraction using solvent mixtures and the salting-out method for improving efficiency of the headspace solid phase extraction [35,36]—with various techniques such as high-performance liquid chromatography, capillary electrophoresis, GC-FID, or GC with mass spectrometry (GC-MS) [36,37,38,39]. Derivatization strategies have also been employed for the quantification of free fatty acids [40]. However, extractions and derivatizations can be time-consuming and require reagents which may be difficult to handle, and salts can be abrasive which may reduce method sensitivity, particularly when analytes are present in lower concentrations. Analysis of SCFAs has been problematic on traditional USP G43 and G35 phase capillary columns, resulting in loss of acidic compounds, lack of reproducible response/retention times, and poor elution profiles with high detection limits. For example, Eberhart II et al. reported a simplified method for the quantitation of SCFA in human stool using an Agilent DB-FFAP (nitro terephthalic acid modified PEG, G35 phase) column but was not applicable to detection of formic acid, as no peak was observed in either aqueous or diethyl ether calibration standards [41]. GC-based methods for detection of formic acid can be found in the literature [23,42,43], but all require prior derivatization of the analyte prior to detection. Derivatization steps are less than ideal as they can leave questions regarding derivatization efficiency, which will impact quantitation accuracy. Furthermore, methods requiring derivatization often require multiple, and sometimes complex and time-sensitive, steps and sample manipulation. Hence, a standard, broadly applicable procedure for their analysis in nanoformulations—without requiring these additional steps—is warranted given the emerging use of these materials in a wide range of applications.

Nanotechnology is often used to formulate API to improve therapeutic index, alter biodistribution, and reduce off-target toxicities and is broadly used across many different therapeutic indications, including cancer therapy, COVID vaccination, antimicrobials, and more [44]. Among the most widely used nanotechnology platforms are liposomes, inorganic metal and metal oxide nanoparticles, polymeric nanoparticles, and lipid nanoparticles (LNP) [45,46]. Various efforts have been employed in the synthesis and purification of drug products using nanomaterials, often employing the use of Class 2 and Class 3 volatile organic solvents and SCFAs. For example, the mRNA-LNP drug product approved for vaccination against coronavirus disease (COVID) was manufactured by mixing an aqueous stream of mRNA with an ethanol suspension of lipids using a NanoAssembler^®^, followed by removal of ethanol via tangential flow filtration and sterile filtration [47]. A microfluidic system is also commonly employed in the formulation of polymeric nanoparticles with low volatility RS, and water-miscible organic solvents are often used for antibody–drug conjugates [48,49]. Nanoformulations pose unique challenges for analysis of RS because of their ultra-small particle size, complex matrices, and high surface area which can alter how RS are retained, released and detected, meaning other RS analytical methods for conventional pharmaceuticals may not always be appropriate, at least without modification. Another main challenge with nanoformulation analysis is strong solvent entrapment, whereby RS may become trapped within the nanoparticle interior or core. Furthermore, complex formulation matrices such as polymers, surfactants, stabilizers, targeting ligands, etc., can often interfere with analytical techniques, including HS-GC, by altering solvent partitioning between the sample and the HS. Matrix-dependent HS behavior can differ significantly among the broad field of nanoformulation technologies compared to conventional dosage forms [50].

The aim of this study was to develop and validate a GC procedure which can serve as a standard test method to detect and quantify the most common volatile organic solvents that may be present in drug products formulated using nanotechnology. The validation of an analytical method for analysis of 19 of the most commonly used Class 2 and Class 3 solvents and SCFAs was carried out per ICH and FDA guidance using PerkinElmer’s HS autosampler (Waltham, MA, USA) with a PerkinElmer Clarus^®^ 690 GC (Waltham, MA, USA) coupled with PerkinElmer SQ8T mass spectrometer (Waltham, MA, USA) which can further enhance selectivity and sensitivity of the method.

## 2. Materials and Methods

### 2.1. Chemical Reagents and Samples

Methanol (99.99%), acetone (99.99%), isopropyl alcohol (99.99%), diethyl ether (99.99%), acetonitrile (99.99%), 1-propanol (99.99%), ethyl acetate (99.98%), tetrahydrofuran (99.98%), dichloromethane (99.97%), chloroform (99.9%), 1-butanol (99.93%), tert-butanol (99.90%), pyridine (99.99%), acetic acid (99.8%), butyric acid (99.9%), valeric acid (99.3%), N,N-dimethylformamide (99.99%), and DMSO (99.99%) were purchased from Sigma Aldrich (St. Louis, MO, USA). Ethanol was obtained from PHARMCO (99.99%). Formic acid (99.6%) was purchased from Thermo Fisher Scientific (Ward Hill, MA, USA). Ultra-pure helium (Research grade, purity > 99.999%), research plus grade nitrogen, and zero grade air were purchased from Airgas, Inc. (Frederick, MD, USA). Ultrapure hydrogen gas (Research grade, purity > 99.999%) was generated from a Parker Domnick Hunter Hydrogen Generator (Model 60H). Doxil (Baxter International, Inc., Deerfield, IL, USA) was purchased from the NIH pharmacy (Division of Veterinary, Resources, Bethesda, MD, USA). Empty liposome (without drug loading) was obtained from FormuMax Scientific, Inc. (Sunnyvale, CA, USA). The polymeric nanoformulation (termed TNM) was generously provided by Professors Ronit Satchi-Fainaro, Tel Aviv University, and Helena Florindo, University of Lisbon. The cross-linked polymer formulation (termed CLP) was generously provided by Privo Technologies (Peabody, MA, USA). The dendrimer nanoconjugate (termed DM) was generously provided by Professor Chi Zhang, University of Nebraska Medical Center.

### 2.2. Instrumentation

A Clarus 690 gas chromatograph equipped with a flame ionization detector, a Turbomatrix HS-40 Trap Headspace autosampler (HST40) and Clarus SQ8 MS (Waltham, MA, USA) in electron ionization were used. Two GC columns—an Elite-624 Crossbond 6% cyanopropylphenyl-94% dimethylpolysiloxane capillary column (0.32 mm ID × 30 m with a 1.8 µm layer) and an Elite-1 100% dimethylpolysiloxane column (0.32 mm ID × 30 m with a 1.8 µm layer)—were used to compare separation efficiencies for the high volatility RS. A DB-Fatwax ultra-inert column (0.25 mm ID × 30 m with a 0.25 µm layer, Agilent Technology (Santa Clara, CA, USA) was used for SCFA analysis, and an Elite-5MS column (15 m × 0.25 mm × 0.25 µm) was used to confirm the target compounds by mass spectrometry. A 2 mm straight-through glass liner (PerkinElmer, Waltham, MA, USA) without wool was used for HS-GC injections, 2 mm split-liner with wool was used for direct injections, and a straight-liner ultra inert split/glass wool liner was used for SCFA injections. HS autosampler 20 mL and 2 mL GC vials were used (PerkinElmer). TotalChrom Navigator (TNav, PerkinElmer, version 6.3.4.0700) and TurboMass software (PerkinElmer, version 6.1.2) were used to control the instrument, data acquisition, processing, and reporting. The final optimized HS-GC and mass spectrometric conditions are presented in Table 1.

### 2.3. Standard and Sample Preparation

Stock solutions of the solvent standards were prepared by dissolving the analytical reference standard in their respective diluents (DMSO for high volatility analytes and methanol for low volatility analytes and SCFA). An appropriate amount of each analyte was transferred to a 10 mL volumetric flask (A grade) half-filled with diluent; analytes are volatile and dispensing into dry flasks results in evaporation and loss of analytes, thus skewing the results. Analytes were mixed immediately after adding into diluent and diluted to volume with diluent. Working standard solutions were prepared by transferring an appropriate amount of the analyte’s stock solution to another volumetric flask and diluted to volume with diluent. The working concentrations of analytes were prepared in such a way to reach 100% of concentrations at their corresponding ICH limits. The corresponding ICH limits of analytes are presented in Table 2.

A separate set of standards was also prepared as “check standards” to confirm the reproducibility of the standard preparations. Serial dilution of each analyte was performed and injected to evaluate the limit of detection (LOD) and limit of quantitation (LOQ). In order to obtain the desired separation and proper signal strength, the first set of 14 analytes was separated into two groups: group I (methanol, ethanol, acetone, 2-propanol, acetonitrile, 1-propanol, ethyl acetate, tetrahydrofuran, 1-butanol, and tert-butanol) and group II (diethyl ether, dichloromethane, chloroform, and pyridine), as grouping RS not only improves separation and quantification but also overall method performance. For all analytes, the calibration standards were prepared in the range of 25% to 150% (linearity level: 25%, 50%, 75%, 100%, 125% and 150% of analyte’s ICH limit) by diluting the stock solutions to 6–8 concentration levels. The various RS have differences in volatility, with each analyte having a different sensitivity to the flame ionization detector; therefore, these concentration levels may be different for each RS.

To evaluate the spike recovery of each analyte, a commercial empty liposome (FormuMax, Inc., Sunnyvale, CA, USA) was used as a sample matrix. The empty liposome serves as a model for evaluating spike recovery in a nanoparticulate system susceptible to partitioning of the RS into the lipid bilayer, aqueous core, or head group interface. Samples were weighed in a GC vial then diluted with appropriate diluent. For analysis, 1 mL of standard solution was pipetted into a GC vial and sealed immediately with a Teflon-lined septum and aluminum crimp cap (PerkinElmer, Waltham, MA, USA). The suggested injection sequence for sample analysis is as follows: diluent as blank, five injections of working standards to evaluate system suitability, check standard, and one bracketing standard injection after 12 injections of samples.

### 2.4. Method Validation

Analytical method validation was performed following ICH and FDA guidelines [51,52]. The assessed criteria were linearity, spike recovery, method precision, analyte stability, specificity, LOD, and LOQ.

#### 2.4.1. Linearity

The linearity of an analytical method validation procedure is defined as the test results within a given range which are directly proportional to the analyte concentration in the sample. Per ICH and USP guidelines, linearity is determined from reporting level to 120% of the ICH limit level [51,53]; an extended linearity range was established in the current study. A calibration curve was constructed using the least squares regression on the analyte peak area against the analyte concentration, in the range of LOQ to 150% of ICH limits implementing the linear fit.

#### 2.4.2. Limit of Detection (LOD) and Limit of Quantitation (LOQ)

The lowest amount of analyte which can be detected in a sample—but not necessarily quantitated as an exact value—is termed LOD, while LOQ is the lowest amount of analyte which can be quantitated with acceptable accuracy and precision. The common approach to determine LOD is 3.3σ/S and LOQ is 10σ/S where σ is the standard deviation of the response and S is the slope of the calibration curve. The signal to noise ratio should be in the range of 3:1 for LOD and 10:1 for LOQ. To establish the LOQ, serial dilution of the working standard solutions was performed to determine the lowest concentration of each analyte that provided a signal-to-noise ratio of 10 or greater. Six injections were made to calculate a relative standard deviation (%RSD) of the response. Per ICH guidance, the %RSD of the injections (n = 6) should be ≤10%.

#### 2.4.3. Specificity

According to ICH Q2(R2), specificity is the ability to unequivocally assess the analyte in the presence of other components which may be present [51,52]. Blank injections and injections of the empty liposome sample were performed to evaluate the presence of any interfering impurities eluting with the analytes of interest.

#### 2.4.4. Spike Recovery, Method Precision and System Precision

The accuracy of an analytical method is the closeness of agreement between the accepted reference value and the observed value. This trueness of the result is quantitatively expressed in terms of bias. Therefore, determination of accuracy allows for estimating the extent to which systematic errors affect a particular method. The accuracy/spike recovery of the method was evaluated using an empty liposome (FormuMax) at the practical limit of quantitation (PLOQ), 50%, and 150% of the analyte’s corresponding ICH limit covering the calibration range (chloroform recovery was performed at the 50%, 100%, and 150% levels). The samples were analyzed as triplicates at each level, and the data was reported as percent bias and percent recovery. Two additional samples, a negative control sample (without spiking of analytes) and a spike control sample (spiked at the 100% ICH limit without matrix), were also prepared for comparison. Method precision was performed at the 100% level, and the coefficient of variation (%CV) was used to evaluate the precision. The ICH acceptance criteria were in the range of 15% (spike recovery) and coefficient of variance was <15.0% (method precision). System precision was also evaluated by injecting working standards of the analytes (n = 6).

#### 2.4.5. Analyte Stability

Analyte sample stability is one of the key analytical parameters to evaluate method efficiency. It is a time period over which the solution maintains accurate concentration within a defined percentage difference from the initial concentration. The samples were prepared (n = 2) by spiking the analytes at the 100% ICH limit level and evaluated for stability at two time points, 24 and 48 h.

#### 2.4.6. Method Application

The clinical drug product Doxil (PEGylated liposomal doxorubicin) and preclinical nanotechnology-based drug products TNM (polymeric nanoformulation), CLP (cross-linked polymer formulation), and DM (dendrimer nanoconjugate) were used as model samples for testing residual volatile impurities. Known weights of samples were taken in 20 mL GC vials, diluted to 1 mL volume with DMSO, and crimped with ultra-low bleed polytetrafluoroethylene (PTFE) septa immediately. The samples were vortexed for 5 min and placed into the HS autosampler for detection and quantitation of high volatility RS. For SCFAs (formic acid, acetic acid, butyric acid, and valeric acid), DMSO, and N,N-DMF analyses, the known weight of sample was taken in 2 mL GC vials, diluted to 1 mL with methanol, crimped with ultra-low bleed PTFE septa, vortexed for 5 min, and analyzed using GC-FID with controlled column temperature gradients. The system suitability test, check standard, and calibration standards were prepared per the procedures described above. A PerkinElmer GC Clarus 690 (Waltham, MA, USA) was connected to a PerkinElmer Clarus SQ8 mass spectrometer (Waltham, MA, USA) in electron ionization mode to verify the analytes studied.

## 3. Results

In the present study, 15 common organic solvents and 4 common SCFAs which have been extensively used in the development of nanoformulation drug products and dissolution of API for the synthesis of drug substances were selected for analysis. The organic solvent analytes included acetone, acetonitrile, 1-butanol, chloroform, dichloromethane, diethyl ether, N,N-dimethylformamide, dimethylsulfoxide, ethanol, ethyl acetate, methanol, 1-propanol, 2-propanol, pyridine, and tetrahydrofuran, and the SCFA analytes included acetic acid, butyric acid, formic acid, and valeric acid.

### 3.1. Optimization of Analytical Method

**Figure 1:**
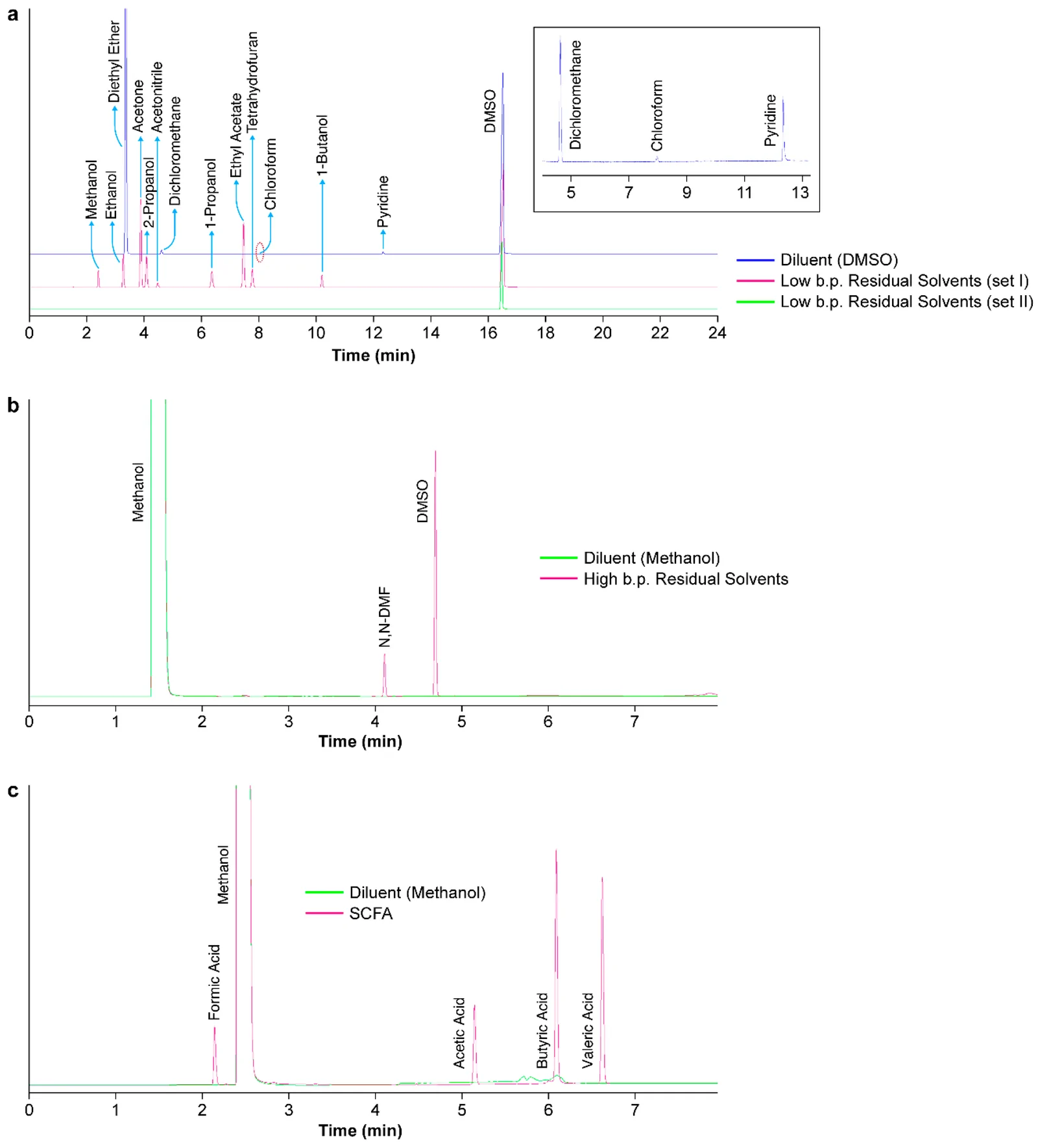
Typical GC-FID chromatograms of the studied RS and SCFA analytes. (**a**) HS-GC-FID chromatograms of 13 low boiling point organic solvent analytes (methanol, ethanol, diethyl ether, acetone, 2-propanol, acetonitrile, dichloromethane, 1-propanol, ethyl acetate, tetrahydrofuran, chloroform, 1-butanol, and pyridine) at the 100% ICH limit level, using DMSO as the diluent. The analytes are shown in two separate chromatograms due to different sensitivities. The inset chromatogram is zoomed to highlight the dichloromethane, chloroform, and pyridine peaks. (**b**) Direct injection GC-FID chromatogram of the high boiling point organic solvent analytes N, N-dimethylformamide and DMSO at the 100% ICH limit level, using methanol as the diluent. (**c**) Direct injection GC-FID chromatograms of the SCFA analytes (formic acid, acetic acid, butyric acid, and valeric acid) at the 100% ICH limit level, using methanol as the diluent.

Several sample-specific parameters were examined as part of the method optimization, as variation on instrument-related parameters such as oven temperature, transfer line temperature, vial pressure, temperature gradient, split ratio, and inlet temperature have been widely investigated [14,54]. For pharmaceutical testing under USP General Chapter <467> and ICH Q3C Guidelines for RS, DMSO, DMF, and water are among the most commonly employed diluents, with the final choice based on a drug product’s solubility and the absence of chromatographic interferences [9,12]. Selection of the appropriate diluent for RS analysis is critical as it can directly affect analyte recovery, chromatographic separation, FID response, and analyte stability. First, two common high boiling solvents, N,N-DMF (boiling point 153 °C) and DMSO (boiling point 189 °C), were evaluated for applicability as the sample diluent for analysis of the high volatility RS. Although there was no interference on retention time of any of the other 13 organic solvent analytes using either solvent (Figure 1), DMSO was selected as the diluent because of its lower toxicity compared to N,N-DMF and because DMSO is a polar aprotic solvent which is stable at high temperatures and possesses a higher capacity for solubilization of many drug delivery nanoformulations. The HS sample equilibration temperature and equilibration time were assessed in a range of 100–130 °C and 5–15 min, respectively. Efficient equilibration for 200 mg nanoformulations was achieved at 120 °C and 8 min. For analysis of SCFA, preliminary experiments were performed with several diluents including methanol, diethyl ether, ethyl acetate, and water (Figure S1, Supplementary Materials). Methanol was found to be the most suitable diluent, as no formic acid peak was detected with diethyl ether and water, and formic acid sensitivity was greatly reduced with ethyl acetate, confirmed by comparing peak area response. Several peaks associated with the ethyl acetate diluent were also observed. Similarly, methanol was used as the diluent for analysis of the low volatility RS, in line with current USP recommendations [55].

Due to complexities associated with the wide variety of nanoplatforms in use, method efficiency was next evaluated by assessing different sample shaking times in the HS sample vial. No differences were observed on analyte response between a vial shaken for 2 min versus a disabled shaking mode when testing the liposomal (Doxil) and polymeric (TNM) nanotechnologies used herein. It is cautioned, however, that due to the vast array of technologies and chemistries used in nanoformulation, this may not be the case for all test samples.

Due to variable polarities of the tested analytes (e.g., the polarity index of chloroform is 2.7 [lowest] versus acetonitrile which is 5.8 [highest]), two GC columns with different stationary phases were used: an Elite 624 Crossbond 6% cyanopropylphenyl-94% dimethylpolysiloxane column and a 100% dimethylpolysiloxane column. System suitability parameters for each peak such as tailing factors, peak resolution, and number of theoretical plates were evaluated. After thorough comparison, the medium polarity column, 6% cyanopropylphenyl-94% dimethylpolysiloxane, was shown to have superior separation efficiency for all studied RS except for SCFAs for which a DB-Fatwax ultra-inert column was used. The optimized HS-GC parameters are presented in Table 1.

### 3.2. Method Validation

#### 3.2.1. Linearity

Method linearity was investigated using 6–9 concentration levels over the approximate range of the PLOQ to 150% of ICH limit for each analyte. The analyte’s ICH option 1 limit, concentration range, linearity equation, and corresponding regression coefficient are shown in Table 2. All analytes demonstrated linearity with a regression coefficient (r2) within the range of 0.9958–0.9998.

#### 3.2.2. Limit of Detection (LOD) and Limit of Quantitation (LOQ)

The LOD and LOQ were determined based on a signal-to-noise response ratio of 3:1 and 10:1, respectively, from serially diluted standard solutions. The established LOQ concentration and %RSD from their LOQ standard injections (n = 6) are presented in Table 3. Notably, formic acid—for which no prior GC method is published which does not require derivatization—has an LOQ of 75 ppm.

#### 3.2.3. Specificity and Sensitivity

The specificity of the method was determined by careful observation of interfering peaks from the diluent and the empty liposomal matrix. (Due to the nature of some nanoformulation preparation procedures, precursor formulations are not always available for screening.) No interference was observed on the retention times of any of the analytes studied. A typical GC chromatogram is presented in Figure S2 (Supplementary Materials). The retention times of the tested analytes are presented in Table 2. The current method demonstrated improved sensitivities for 17 of the 19 tested analytes compared to previous studies (Table 3).

#### 3.2.4. Accuracy and Recovery

Accuracy of the method was evaluated through spike and recovery experiments at the PLOQ, 50% level, and 150% level of ICH limits in a triplicate analysis. The calculated recovery and percent bias at these levels are presented in Table 4. Data from the negative control without spiking and spiked control at 100% ICH limit were also collected for comparison. Recovery was observed between 83.3 and 104.1%. The percentage bias for the 50–150% limit levels was found to be within ±7.4%, while ±16.7% bias was observed for the PLOQ level. The recommended ICH acceptance criteria states the mean recovery for each spiked sample solution should be 80–120% [56]; therefore, these data demonstrate the accuracy of the HS-GC method.

#### 3.2.5. Method Precision, System Precision and System Suitability

Precision was assessed by evaluating the intra-day precision/repeatability of the analysis. The analytes were spiked at the 100% level in the empty liposome formulation, and their recoveries were evaluated. The results are presented by the %RSD of six preparations of each solvent. The intra-day accuracy values (% bias) were within ±6.1 and %RSD were 0.6–7.4% (n = 6), confirming the precision of the optimized method. The analyte chromatograms are presented in Figure 1, and the data are summarized in Table 5. The system precision (injection precision) was also evaluated by injecting working standards (n = 5), and the %RSD was ≤4.1% for all tested analytes with the exception of valeric acid which was 7.4%.

For verification of the system suitability for the acquired data, one set of a separate standard in working concentration range (check standard) was also prepared; the percent recovery of the check standard and bracketing standard was between 98% and 104% throughout the entire analysis (Table S1, Supplementary Materials).

#### 3.2.6. Analyte Stability

The spike recovery of the analytes (n = 2), stored at room temperature, at the 100% level were re-injected at 24 h and 48 h to evaluate analyte stability. The percentage difference of the concentration at 24 h and 48 h versus the initial concentration (C0) were determined and are presented in Table S2 (Supplementary Materials). The percent difference between the initial results (C0) and 24 h results was ≤7.7% and for the 48 h results was ≤9.8%. Of note, this study did not include evaluation of working standard solution stability as several earlier studies have reported RS standard stability in DMSO [21,51].

### 3.3. Analysis of Volatile Impurities in Test Nanoformulations

The optimized methods were applied for detection and quantitation of residual volatile impurities in four different nanoformulations—Doxil, a clinical PEGylated liposome encapsulating doxorubicin, and three preclinical research formulations, a polymeric nanoformulation (TNM), a cross-linked polymer formulation (CLP), and a dendrimer nanoconjugate (DM), all being studied as potential new treatment options for various cancer indications.

**Figure 2:**
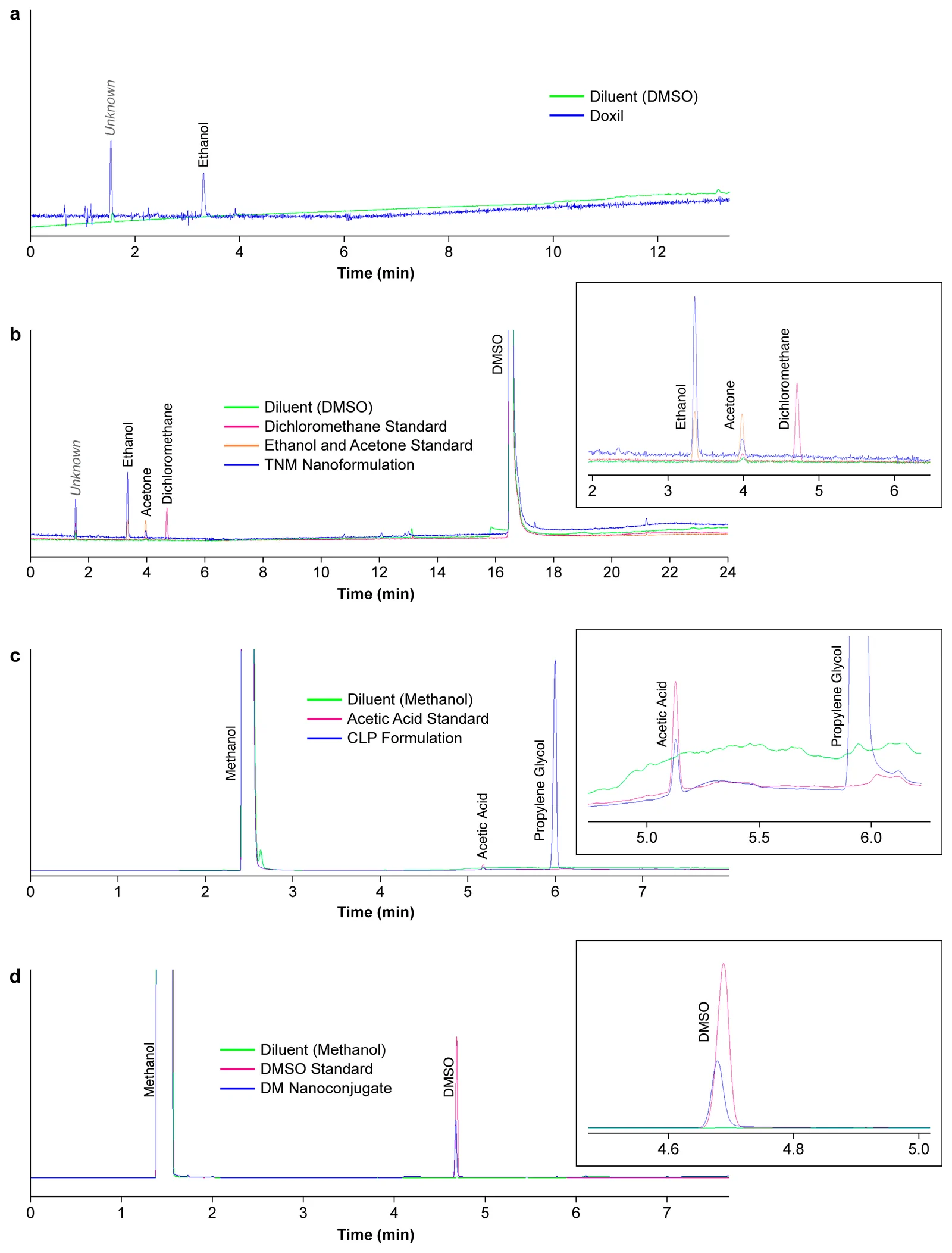
Typical GC-FID chromatograms of test nanoformulations. (**a**) HS-GC-FID chromatogram of the liposomal Doxil formulation showing the presence of ethanol in the test sample. (**b**) HS-GC-FID chromatogram of the TNM nanoformulation showing the presence of both ethanol and acetone in the test sample. The inset chromatogram is zoomed to highlight the presence of ethanol and acetone peaks as well as the absence of the dichloromethane peak in the test sample. (**c**) Direct injection GC-FID chromatogram of the CLP formulation showing the presence of both acetic acid and pyridine. The inset chromatogram highlights the acetic acid in the test sample. (**d**) Direct injection GC-FID chromatogram of the DM nanoconjugate showing the presence of DMSO.

One RS was detected in the commercial Doxil formulation, ethanol. Ethanol is a commonly utilized solvent in the synthesis and purification of liposomes. The amount of residual ethanol in Doxil was found to be 8.64 µg/mL, corresponding to 43 ppm, (Table 6; %RSD = 5.2, n = 3). A typical GC-FID chromatogram of Doxil is shown in Figure 2a.

The TNM nanoformulation was initially screened for dichloromethane, as this solvent was used in production of the material; however, the HS-GC-FID analysis showed no residual dichloromethane up to 0.001% (by weight) of sample concentration (Figure 2b, Table 6). Thus, the purification procedure employed was adequate in removing this solvent to acceptable levels. Interestingly, though, two additional, unexpected RS were detected. Ethanol was detected at 84 ppm (%RSD = 0.3, n = 3), and traces of acetone were detected but were below the LOQ.

For the CLP formulation, acetic acid was used to solubilize the backbone polymer, and indeed, residual acetic acid was present in the formulation at 3241 ppm (Figure 2c, Table 6). The method also detected propylene glycol which was used as part of the diluent system for reconstitution of the formulation prior to administration; this peak was confirmed by GC-MS (Figure S3, Supplementary Materials).

Residual DMSO, which was used to solubilize the API in the DM nanoconjugate, was assessed using the direct injection method and was present in the sample at 7040 ppm (Figure 2d, Table 6). The ICH limit for DMSO is 0.5% or 5000 ppm (Table 2). This result was also confirmed using GC-MS (Figure S4, Supplementary Materials).

## 4. Discussion

The detection and quantitation of RS in nanomedicines have significant importance. These complex formulations possess unique relationships with respect to physicochemical properties and biological effects. Even low levels of some RS can have an effect on a formulation’s physicochemical characteristics, which, in turn, could impart effects on the efficacy and toxicity of the formulation. For example, RS have been shown to affect dissolution rates, molar mass, structure, and water permeability of certain polymers [8]. Given the emerging use of polymers in nanotechnology drug delivery [45,46], detection and quantitation of RS become important not only in the final drug products, but also in the precursor components of the formulation. Even more so, trace levels of some solvents can pose significant safety concerns for drug products being developed for eventual clinical use. Certain Class 2 solvents have concentration limits as low as 50 ppm, while many Class 1 solvents—which may not be used directly in the manufacture of the final nanomedicine drug product but could be employed in the preparation of certain starting materials—are <10 ppm [9]. Given the broad use of solvents in use in the nanomedicine research space, a highly sensitive and broadly applicable method is needed. While GC has been the standard approach for detection and quantification of RS for many years, given the tremendous advances in both nanoformulation and analytical capabilities, a renewed look into the approach was warranted.

Initial activities focused on optimizing separation and detection conditions for low boiling point RS (e.g., methanol, ethanol, diethyl ether, acetone, 2-propanol, acetonitrile, dichloromethane, 1-propanol, ethyl acetate, tetrahydrofuran, chloroform, 1-butanol, tert-butanol, and pyridine). Efforts included selection of a broadly applicable set of diluents, adequate sample resuspension, and analysis of suitable columns. Use of a single diluent for all RS is not possible due to different volatilities and partition coefficients of the many possible RS. The tendency and magnitude of diluent effects depends on the polarity of the analyte solvents as well as the diluents and are further affected by the sample solvation process [57]. In static HS, analyte solvents with polarity values higher than that of the diluent will be strongly trapped in the liquid phase, and hence such solvents will produce low concentration in the gas phase with smaller peak response and vice versa. DMSO was found to be a suitable diluent for analysis of many low boiling point RS since it is more stable at high temperature (b.p. 189 °C) and did not have significant interference in the elution range of the target analytes. Note that HS equilibrium efficiency can only be obtained if the HS oven temperature is lower than the boiling temperature of the solvent.

Method performance was evaluated using different shaking modes for the HS sample vial. Vial shaking during incubation produces a large exchange surface between the liquid and vapor phases; this enhances homogenous migration of the analytes in the headspace, thereby speeding up equilibration and improving method efficiency, especially for analytes with low partition coefficients. Although no differences in analyte response were observed for the two test nanoformulations described herein, this can be an important added step for many formulation types and thus should be considered when analyzing other complex formulations.

The retention of polar solvents is much stronger in mid-polar columns at high temperatures which provide high separation between solvents with minor differences in boiling points. As shown in Figure 1 and Table 2, most of the analyzed RS are well separated from each other using the Elite 624 column. The closely eluting analyte pairs such as ethanol/diethyl ether and acetonitrile/dichloromethane were further examined by comparing the Elite 624 stationary phase (equivalent to USP phase G43) 6% cyanopropylphenyl-94% dimethylpolysiloxane to an Elite 1 stationary phase (equivalent to USP phase G2) 100% dimethylpolysiloxane. Under the same chromatographic conditions, the peak resolution was found to decrease to 0.7 from 1.0 with the G2 phase column for the acetonitrile/dichloromethane pair, clearly indicating the superiority of 6% cyanopropylphenyl-94% dimethylpolysiloxane column. The standard G43 phase is the USP recommended column for the analysis of polar solvents such as methanol and acetonitrile and mid-polar to less polar solvents such as acetone, pyridine, ethanol, ether, 1-propanol, 2-propanol, 1-butanol, chloroform, ethyl acetate, THF, and dichloromethane [9]. However, the critical solvent pair ethanol and diethyl ether were incompletely resolved, with peak resolution ≥ 1.0, which is the practical limitation of the method in its current form. The GC-FID method by Bernardoni et al. was able to resolve the critical pair of ethanol/diethyl ether with a peak resolution < 1.0 by using hydrogen as the carrier gas. For samples requiring the analysis of such critical pairs, improved peak resolution can generally be attained by tuning the carrier gas flow, optimizing the pressure programming, and adjusting the column’s gradient parameters to enhance separation.

Next, conditions were explored for optimal separation and detection of high boiling point solvents, e.g., N,N-DMF and DMSO. Based on our preliminary evaluation and method validation data (Figure 1b), the analytes DMSO and N,N-DMF were well resolved using the Elite 624 stationary G43 phase, proving to be suitable for efficient separation of the two analytes. Both analytes exhibited baseline resolution, eluted from the column within 8 min, and had suitable peak shape. Thus, this method required no further optimization for the two studied analytes. Other high boiling RS such as N,N-dimethylacetamide, ethylene glycol, N-methyl-2-pyrrolidone propylene glycol, etc., can also be included for analysis using this procedure.

Multiple analytical methods have been reported for analysis of SCFAs, and GC-FID has been used to detect low molecular weight carboxylic acids in their free form—with the notable exception of formic acid, an important intermediate used in the synthesis various API and nanoformulations. Normally, derivatization to methyl formate or another similar analyte is required to detect formic acid using FID [23,42,43]. The practical difficulty in separation is the influence of the acid response on conditioning time/temperature which is not stable after heating the column. After heating and conditioning the column at low temperature, acid recovery improves, but this effect is immediately lost once heat is applied again. To compensate for this, a DB-Fatwax ultra-inert (UI) column using methanol as the diluent was employed to improve the separation performance for formic acid. It is noted that other tested diluents either failed to show a detectable formic acid peak (e.g., diethyl ether and water) or showed greatly reduced sensitivity (ethyl acetate) (Figure S1, Supplementary Materials). Moreover, this method allowed for simultaneous detection of other SCFAs such as acetic acid, butyric acid, and valeric acid, showing a clear advantage over other previously published methods [41]. Agilent reports used a similar DB-Fatwax-UI capillary column separation phase for formic acid; however, they report poor detection abilities using FID and required mass spectrometry for adequate detection [58]. To the best of our knowledge, there have been no methods reported in the literature for simultaneous determination of trace amounts of formic acid, acetic acid and other SCFAs without derivatization.

The optimized parameters for GC detection of low boiling point, high boiling point, and SCFA are summarized in Table 1. After optimization of these parameters, the method was validated according to ICH and FDA guidance, to include linearity (Table 2), accuracy and spike recovery (Table 4), method precision (Table 5), analyte stability (Table S2), specificity, LOD, and LOQ (Table 3) [51,52]. All parameters were within the specified acceptance criteria. Furthermore, the current method showed better sensitivity/lower LOQ compared to previously published reports using similar instrumental conditions (Table 3) [13,22,30].

The next objective of this study was to test the optimized methods for the detection and quantitation of RS in various nanoformulation test samples. As liposomes and polymeric nanoparticles are two of the most commonly used platforms [45,46], one of each was selected for inclusion in this study. The evaluation of RS in the commercial Doxil formulation revealed the presence of residual ethanol at 43 ppm (% RSD = 5.2, n = 3) (Figure 2a, Table 6). This level of residual ethanol is considered safe from both a regulatory and patient safety perspective. Moreover, assessment at this level of sensitivity is meaningful and helps in assessing the potential impact of RS on the physicochemical properties of nanomaterials.

Upon analysis of the TNM preclinical polymeric nanoformulation, no residual dichloromethane—which was used in production of the formulation—was detected up to 0.001% of the sample concentration (ICH limit: <0.06% or 600 ppm). Thus, the employed purification protocol was adequate in reducing residual dichloromethane to acceptable levels. Interestingly, however, the method detected the presence of residual ethanol (84 ppm) and traces of acetone (<5 ppm) in the sample (Figure 2b, Table 6). Although not used directly in production of the formulation, ethanol was used in the laboratory to clean glassware, and acetone was used in the production of one of the starting polymeric material components. Thus, the high sensitivity of this method, combined with its ability to detect a broad range of analytes in a single run, was able to detect RS which were not suspected to be contributing contaminants in the final formulation. Given the broad analyte range, high sensitivity, and rapid and facile nature of the HS-GC method, it can be a valuable addition to the set of assays used in lot release testing.

Additional preclinical formulations CLP (cross-linked polymer formulation) and DM (dendrimer nanoconjugate) were analyzed for residual acetic acid and DMSO, respectively, used to solubilize the backbone polymer (CLP) and API (DM) in the respective drug products. CLP analysis showed residual acetic acid at 3241 ppm (Figure 2c, Table 6). The method was also able to detect propylene glycol, a component of the diluent system (Figure 2c; Figure S3, Supplementary Materials), indicating an additional robustness of the method. Analysis of the DM nanoconjugate showed residual DMSO at 7040 ppm. The ICH acceptable level of DMSO is 5000 ppm; thus, this method was able to demonstrate a need for improved solvent reduction/removal processes before advancing further in the developmental pipeline. Both results were further confirmed using GC-MS, adding confidence to compound identification.

The procedures described herein for high volatility (low boiling point) RS, low volatility (high boiling point) RS, and SCFAs are more sensitive than previously published reports (Table 3) and can be applied without difficulty, even for the analysis of small amounts of samples (~<100 mg). In comparison, Stolarczyk et al. developed a method for quantitative analysis of semi-volatile solvents such as DMSO and acetic acid, and obtained LOQ values of 20 µg/mL and 46 µg/mL, respectively [33]. Several others have reported methods for DMSO quantitation as well: Tian et al. reported an LOQ of 49 ppm [34], Somuramasami et al. reported an LOQ of 50 ppm using a static HS-GC method [32], and Mana Kialengila et al. reported an LOQ of 28 ppm using full evaporation HS-GC [31]. The present method is more sensitive for both analytes, with an LOQ of 4.0 µg/mL or 20 ppm for DMSO and 5 µg/mL or 25 ppm for acetic acid. In fact, sensitivities were improved for all studied analytes with the exception of pyridine and N,N-DMF. The optimized GC methods developed herein for both low and high volatility solvents have been made available in step-by-step protocol format for easy adaptation in the nanomedicine research community [59,60,61].

## 5. Conclusions

The GC method described in this study was validated for detection and quantitation of 19 common volatile organic solvents and SCFA per ICH guidelines, and the method was demonstrated to be sensitive, linear, specific, accurate, stable, and precise. The rationale for selecting the column, diluent, and other HS parameters was described, resulting in a method with a much broader detection range, shorter HS equilibration time, and higher sensitivity compared to previously published methods. The high sensitivity makes this method valuable for not only analysis of known process impurities but other common impurities that may be present from handling steps, cleaning of glassware and instruments, etc. The method was also shown to be applicable for the quantitative analysis of SCFAs, including formic acid, without the need for added extraction or derivatization steps—an important distinction, as the added sample handling steps and efficiency of the extraction/derivatization steps can impact quantitation accuracy. Overall, this method was demonstrated to be a straightforward and broadly applicable approach suitable for high throughput analysis of many commonly used residual volatile impurities found in API, polymers and polymer–drug conjugates, lipids, dendrimers, and other species used in the production of nanomedicine formulations.

## Figures and Tables

**Table 1:** HS-GC-FID and MS instrument conditions.

Parameters	Optimized Conditions
**HS 40 Trap**	
Oven Temperature	120 °C
Needle Temperature	120 °C
Carrier Gas/Pressure	Helium/15 psi
Thermostatting Time	2 min
Pressurization Time	0.5 min
Withdraw Time	0.2 min
Operating Mode	Constant
Shaking/Hi PSI Inject	Disabled
Transfer line Temperature	120 °C
Injection Time	0.04 min
GC cycle	27.5 min
**GC, Clarus 690 PE (HS-GC Method for High Volatility Analytes)**	
Carrier Gas/Pressure	Helium/10 psi
Column	Elite 624 Crossbond 6% cyanopropylphenyl-94% dimethylpolysiloxane
Temperature Gradient	40 °C initial, hold for 6 min; ramp to 200 °C @ 10 °C/min, hold for 3 min
Injector Temperature	200 °C
Split Ratio	None
Detector Temperature	250 °C
Hydrogen Flow	35 mL/min
Air Flow	400 mL/min
Run Time	25 min
**GC, Clarus 690 PE (Direct Injection Method for Low Volatility Analytes and SCFA)**	
Carrier Gas/Pressure	Helium/10 psi
Column	Elite 624 Crossbond 6% cyanopropylphenyl-94% dimethylpolysiloxane OR DB-Fatwax ultra-inert column (for SCFA)
Temperature Gradient	70 °C initial, hold for 1 min; ramp to 130 °C @ 50 °C/min, hold for 1 min; ramp to 220 °C @ 50 °C/min, hold for 3 min
Injector Temperature	220 °C
Injection Volume	1 µL
Split Ratio	1:20
Pre/post injection wash	2/2
Detector Temperature	250 °C
Hydrogen Flow	45 mL/min
Air Flow	450 mL/min
Run Time	8 min
**MS, Clarus SQ8T Parameters**	
Mass Range (*m*/*z*)	32–200
Source Temperature	225 °C
Column	Elite-5MS capillary column
Source Temperature	225 °C
Inlet Line Temperature	225 °C
Ionization	Electron impact
Solvent Delay	0–1 min
Multiplier	1362 V

**Table 2:** Linearity of class 2 and class 3 solvents. Analytes are divided by high volatility (top), low volatility (middle), and SCFA (bottom) and are listed in order of increasing retention time within each section.

Analytes	ICH Option 1 Limit (ppm)	Equation	Retention Time (min)	Conc. Range (µg/mL)	Regression Coefficient (R^2^)
Methanol	3000	y = 3.1507x − 9.0639	2.92	15.01–900.51	0.9998 (n = 8)
Ethanol	5000	y = 3.9989x − 21.617	3.32	26.76–1605.44	0.9998 (n = 8)
Diethyl Ether	5000	y = 39.008x − 15.646	3.37	1.02–1524.50	0.9998 (n = 7)
Acetone	5000	y = 13.498x − 7.066	3.91	23.69–1421.40	0.9998 (n = 7)
2-Propanol	3000	y = 4.8613x − 19.224	4.19	25.53–1531.65	0.9998 (n = 8)
Acetonitrile	410	y = 5.8344x − 0.7549	4.53	2.46–147.59	0.9996 (n = 8)
Dichloromethane	600	y = 3.2681x − 0.793	4.60	6.88–206.34	0.9991 (n = 7)
1-Propanol	5000	y = 2.887x − 8.921	6.47	25.16–1509.30	0.9998 (n = 8)
Ethyl Acetate	5000	y = 10.44x − 9.9184	7.49	25.42–1525.34	0.9989 (n = 7)
Tetrahydrofuran	720	y = 17.663x + 0.5539	7.80	4.31–258.56	0.9998 (n = 7)
Chloroform	60	y = 1.6261x − 0.024	7.97	3.05–18.29	0.9991 (n = 6)
1-Butanol	5000	y = 1.6398x − 8.0795	10.27	25.33–1519.94	0.9998 (n = 8)
Pyridine	200	y = 4.0586x + 0.3647	12.34	2.38–71.40	0.9986 (n = 6)
N,N-DMF	880	y = 2.6324x − 48.211	4.10	2.46–246.22	0.9958 (n = 9)
DMSO	5000	y = 2.8678x − 63.105	4.70	4.00–1200.48	0.9984 (n = 9)
Formic Acid	5000	y = 0.4245x − 19.235	2.15	150.00–1500.00	0.9969 (n = 9)
Acetic Acid	5000	y = 0.5789x − 0.147	5.17	50.00–1500.00	0.9995 (n = 9)
Butyric Acid	5000	y = 1.5907x + 15.491	6.08	50.00–1500.00	0.9985 (n = 9)
Valeric Acid	5000	y = 1.4765x − 6.233	6.61	50.00–1500.00	0.9991 (n = 9)

Note: x is the concentration of each residual solvent, and y is the peak area in the linear regression fit.

**Table 3:** Limit of detection (LOD) and limit of quantitation (LOQ) for studied Class 2 and Class 3 solvents and comparison of sensitivity.

Analytes	LOD (µg/mL)	LOQ (µg/mL)		LOQ with Respect to Sample Conc. (ppm)	%RSD (n = 6)	References
		Current Work	Previous Reports			
Methanol	0.75	2.10	7.91, 23.7	11	2.9	[22,30]
Ethanol	1.34	3.75	7.9, 7.9	19	2.6	[22,30]
Diethyl Ether	0.51	1.02	7.06	5	1.1	[22]
Acetone	1.18	3.32	7.91, 4.7	17	1.0	[22,30]
2-Propanol	1.28	3.57	7.85, 7.8	18	4.0	[22,30]
Acetonitrile	0.34	2.46	7.86, 11.8	12	5.9	[22,30]
Dichloromethane	3.44	6.88	13.25, 19.9	34	2.3	[22,30]
1-Propanol	1.26	3.52	8.04, 12.0	18	3.9	[22,30]
Ethyl Acetate	1.27	3.56	9.02, 5.4	18	1.7	[22,30]
Tetrahydrofuran	0.60	2.80	8.89, 4.4	9	2.4	[22,30]
Chloroform	3.05	6.10	7.40	30	6.9	[16]
1-Butanol	1.27	3.55	8.10, 24.3	18	3.7	[22,30]
Pyridine	1.30	2.38	1.21	12	9.8	[14]
DMSO	1.50	4.00	20.0, 25.0	20	1.7	[32,33]
N,N-DMF	0.82	2.46	0.68	12	1.4	[16]
Formic Acid	5.05	15.00	-	75	0.80	-
Acetic Acid	1.50	5.00	46.0	25	5.5	[33]
Butyric Acid	1.50	5.00	12.0	25	4.4	[41]
Valeric Acid	1.50	5.00	12.0	25	8.9	[41]

**Table 4:** Accuracy/recovery values of residual solvent analytes.

Analytes	Level n = 3 (%)	Amount Added, µg/mL (µ_o_)	Amount Recovered, µg/mL ($\overset{-}{X}$)	Bias = ($\overset{-}{X}$ − µ_o_)	%Bias = ($\overset{-}{X}$ − µ_o_/µ_o_) ∗ 100	% Recovery = ($\overset{-}{X}$/µ_o_) ∗ 100
Methanol	150	918.5	921.4	2.9	0.3	100.3
	50	306.2	314.1	7.9	2.6	102.6
	PLOQ	6.1	6.2	0.1	1.6	101.6
Ethanol	150	1532.9	139.8	6.9	0.5	100.5
	50	511.0	528.3	17.3	3.4	100.3
	PLOQ	10.2	10.2	0	0	100.0
Diethyl Ether	150	1456.7	1457.5	0.8	0.1	100.1
	50	485.6	452.9	−32.7	−6.7	93.3
	PLOQ	4.9	4.5	−0.5	−9.2	90.8
Acetone	150	1498.2	1503.5	5.3	0.4	100.4
	50	499.4	513.7	14.3	2.9	102.9
	PLOQ	10.0	10.2	0.2	2.0	102.0
2-Propanol	150	1763.8	1774.4	10.6	0.6	100.6
	50	587.9	612.1	24.2	4.1	104.1
	PLOQ	11.8	11.3	−0.5	−4.3	95.7
Acetonitrile	150	124.2	123.6	−0.6	−0.5	99.5
	50	41.4	42.3	0.9	2.2	102.2
	PLOQ	1.0	0.9	−0.1	−10.0	90.0
Dichloromethane	150	172.8	177.2	4.4	2.6	102.5
	50	57.6	57.4	−0.2	−0.4	99.7
	PLOQ	5.8	5.7	−0.1	−1.7	98.3
1-Propanol	150	1589.7	1598.7	9.0	0.6	100.6
	50	529.9	502.3	−27.6	−5.2	94.8
	PLOQ	10.6	9.9	−0.7	−6.6	93.4
Ethyl Acetate	150	1498.5	1510.9	12.4	0.8	100.8
	50	499.5	476.0	−23.5	−4.7	95.3
	PLOQ	10.0	9.7	−0.3	−3.4	96.6
Tetrahydrofuran	150	252	252.7	0.7	0.3	100.3
	50	84	86.6	2.6	3.1	103.1
	PLOQ	1.7	1.7	0	−1.2	98.8
Chloroform	150	22.3	22.0	−0.32	−1.4	98.6
	50	7.4	6.9	−0.5	−6.8	93.2
1-Butanol	150	1521.1	1547.1	26	1.7	101.7
	50	507.0	469.5	−37.5	−7.4	92.6
	PLOQ	10.1	9.91	−0.2	−1.9	98.1
Pyridine	150	58.8	56.5	−2.3	−3.9	96.1
	50	19.6	19.1	−0.5	−2.6	97.4
	PLOQ	7.8	6.5	−1.3	−16.7	83.3
N,N-DMF	120	246.2	254.2	8.0	3.2	103.2
	80	164.1	167.0	2.9	1.8	101.8
	PLOQ	3.1	2.9	−0.2	−7.2	92.8
DMSO	120	1200.5	1229.3	28.8	2.4	102.4
	80	800.3	819.0	18.7	2.3	102.3
	PLOQ	15.0	14.0	−1.0	−6.7	93.4
Formic Acid	120	1238.2	1146.5	−91.8	−7.4	92.6
	80	825.5	784.8	−40.6	−4.9	95.1
	PLOQ	51.6	51.9	0.3	0.6	100.6
Acetic Acid	120	1220.4	1269.0	48.6	4.0	104.0
	80	813.6	817.3	3.7	0.5	100.5
	PLOQ	50.9	49.2	−1.7	−3.3	96.8
Butyric Acid	120	1286.2	1271.2	−15.0	−1.2	98.8
	80	857.5	871.7	14.2	1.7	101.7
	PLOQ	53.6	46.6	−7.0	−13.0	87.0
Valeric Acid	120	1177.5	1189.7	12.2	1.0	101.0
	80	785.0	814.8	29.8	3.8	103.8
	PLOQ	49.1	45.6	−3.4	−7.0	93.0

**Table 5:** Method precision of residual solvent analytes.

Analytes	Conc. Added (µg/mL)	Conc. Recovered (µg/mL)	Mean Recovery (%)	% Bias	%RSD
Methanol	612.3	613.1	100.1	0.1	1.4
Ethanol	1021.9	1027.9	100.6	0.6	1.1
Diethyl Ether	971.1	947.1	97.5	−2.5	1.6
Acetone	998.8	1002.4	100.4	0.4	0.7
2-Propanol	1075.9	1186.2	100.9	0.9	1.0
Acetonitrile	82.8	83.5	100.8	0.9	2.6
Dichloromethane	115.2	115.6	100.3	0.4	0.6
1-Propanol	1059.8	1073.4	101.3	1.3	1.8
Ethyl Acetate	999.0	1008.0	100.9	0.9	0.8
Tetrahydrofuran	168	168.8	100.5	0.5	1.0
Chloroform	14.9	15.3	102.7	2.7	3.8
1-Butanol	1014.1	1019.9	100.6	0.6	1.5
Pyridine	39.2	39.4	100.5	0.5	1.4
N,N-DMF	225.7	218.6	96.8	−3.2	2.2
DMSO	1100.4	1078.8	98.0	−2.0	2.7
Formic Acid	1031.9	969.1	93.9	−6.1	4.1
Acetic Acid	1070.0	1015.5	99.9	−0.2	3.4
Butyric Acid	1071.8	1102.8	102.9	2.9	2.6
Valeric Acid	981.2	1015.2	103.5	3.5	7.4

**Table 6:** Nanoformulation RS analysis.

Nanoformulation	RS Detected	Concentration (ppm)	ICH Option 1 Limit (ppm)
Doxil	Ethanol	43	5000
TNM	Dichloromethane	Not Detected (<0.001% of sample conc)	600
TNM	Ethanol	84	5000
CLP	Acetic acid	3241	5000
DM	DMSO	7040	5000

Note: TNM, polymeric nanoformulation; CLP, cross-linked polymer nanoformulation; DM, dendrimer formulation.

## Data Availability

The original contributions presented in this study are included in the article/Supplementary Material. Further inquiries can be directed to the corresponding author.

## Supplementary Materials

The following supporting information can be downloaded at: https://www.mdpi.com/article/10.3390/mps9040110/s1, Table S1: System suitability; Table S2: Evaluation of analyte sample stability; Figure S1: Typical GC-FID chromatograms of the SCFA analytes diluted in (a) diethyl ether (b) ethyl acetate and (c) water; Figure S2: Typical GC-FID chromatograms of the DMSO diluent and FormuMax empty liposome diluted in DMSO; Figure S3: (a) Total ion chromatogram of the CLP formulation, showing the presence of acetic acid and propylene glycol, (b) molecular ion spectrum of the acetic acid peak, and (c) molecular ion spectrum of the propylene glycol peak; Figure S4: (a) Total ion chromatogram and (b) molecular ion spectra of the DMSO standard at 88 ppm, and (c) total ion chromatogram and (d) molecular ion spectra of the DM nanoconjugate.

## Abbreviations

The following abbreviations are used in this manuscript:

API	active pharmaceutical ingredient
CLP	cross-linked polymer
CV	coefficient of variation
DM	dendrimer nanoconjugate
DMSO	dimethyl sulfoxide
EP	European Pharmacopeia
FDA	Food and Drug Administration
FID	flame ionization detector
HS-GC	headspace-gas chromatography
ICH	International Council for Harmonization of Technical Requirements for Pharmaceuticals for Human Use
LNP	lipid nanoparticles
LOD	limit of detection
LOQ	Limit of quantitation
MHRA	Medicines and Healthcare products Regulatory Agency
MS	mass spectrometer
N,N-DMF	N,N-dimethylformamide
PDE	permitted daily exposure
PLOQ	practical limit of quantitation
PPM	parts per million
PTFE	polytetrafluoroethylene
RS	residual solvent
RSD	relative standard deviation
SCFA	short chain fatty acid
TGA	Therapeutic Goods Administration
THF	tetrahydrofuran
TNM	polymeric nanoformulation
USP	United States Pharmacopeia
