# Supplementary material for: Gas Chromatography Method for Quantitation of Residual Solvent Impurities in Nanoformulations
Source: Methods Protoc. 2026 Jul 23;9(4):110. doi: 10.3390/mps9040110 (PMC13515910; doi:10.3390/mps9040110)
Supplement: Supplementary file 1 [file mps-09-00110-s001.zip › mps-4387458-supplementary.pdf]

Supplementary Materials for

Gas Chromatography Method for Quantitation of Residual Solvent Impurities in Nanoformulations

Krishna Kattel, Rachael M. Crist and Jeffrey D. Clogston*

Nanotechnology Characterization Laboratory, Cancer Research Technology Program, Frederick National Laboratory for Cancer Research, Frederick, Maryland, USA

Corresponding author:
Jeffrey D. Clogston, clogstonj@mail.nih.gov

Table S1……………………………………………………………………………………………2
Table S2……………………………………………………………………………………………3
Figure S1…………………………………………………………… ……………………………4
Figure S2…………………………………………………………… ……………………………5
Figure S3…………………………………………………………… ……………………………6
Figure S4…………………………………………………………… ……………………………7

Table S1. System suitability.

Analytes	System Precision (%RSD, 5 injections)	Check Standard Recovery (%)	Bracketing Standard Recovery (%)
Methanol	0.39	101.4	99.4
Ethanol	0.95	99.2	100.5
Diethyl Ether	0.90	99.4	101.0
Acetone	1.9	101.7	102.7
2-Propanol	1.7	101.3	102.4
Acetonitrile	1.2	103.9	98.1
Dichloromethane	1.1	100.4	102.0
1-Propanol	1.4	100.0	101.9
Ethyl Acetate	1.6	101.6	102.1
Tetrahydrofuran	1.1	101.3	102.9
Chloroform	1.8	101.2	102.2
1-Butanol	1.5	100.8	102.5
Pyridine	3.8	98.8	102.9
N,N-DMF	0.88	99.4	101.3
DMSO	1.1	101.6	100.4
Formic Acid	1.2	100.5	103.3
Acetic Acid	0.66	100.6	102.5
Butyric Acid	1.3	102.0	99.6
Valeric Acid	0.91	99.8	100.2

Table S2. Evaluation of analyte sample stability.

Analytes	Run	Initial Amount (mg/mL)	Amount after 24 h (mg/mL)	% Difference from Initial	Amount after 48 h (mg/mL)	% Difference from Initial
Methanol	I	0.6270	0.5849	6.9	0.5732	8.9
	II	0.6231	0.5883	5.7	0.5777	7.6
Ethanol	I	1.0403	0.9889	5.1	0.9663	7.4
	II	1.0412	0.9934	4.7	0.9723	6.8
Diethyl Ether	I	0.9463	0.9065	4.3	0.8892	6.2
	II	0.9331	0.9132	2.2	0.8889	4.9
Acetone	I	1.0126	0.9625	5.1	0.9639	4.9
	II	1.0107	0.9724	3.9	0.9726	3.8
2-Propanol	I	1.2011	1.1370	5.5	1.1014	8.7
	II	1.1952	1.1452	4.3	1.1031	8.0
Acetonitrile	I	0.0843	0.0780	7.7	0.0771	8.9
	II	0.0843	0.0808	4.3	0.0796	5.8
Dichloromethane	I	0.1164	0.1140	2.1	0.1123	3.6
	II	0.1158	0.1143	2.1	0.1125	2.9
1-Propanol	I	1.0860	1.0232	6.0	0.9906	9.2
	II	1.0849	1.0299	5.2	1.0039	7.8
Ethyl Acetate	I	1.0169	0.9563	6.1	0.9424	7.6
	II	1.0151	0.9662	4.9	0.9415	7.5
Tetrahydrofuran	I	0.1695	0.1622	4.4	0.1622	4.4
	II	0.1704	0.1641	3.7	0.1606	5.9
Chloroform	I	0.0149	0.0140	6.8	0.0139	7.6
	II	0.0155	0.0144	6.9	0.0143	7.5
1-Butanol	I	1.0424	0.9772	6.5	0.9561	8.6
	II	1.0108	0.9857	2.5	0.9613	5.0
Pyridine	I	0.0390	0.0370	5.2	0.0361	7.6
	II	0.0404	0.0379	6.3	0.0366	9.8
N,N-DMF	I	0.2240	0.2100	6.4	0.2195	2.0
	II	0.2239	0.2191	2.1	0.2196	1.9
DMSO	I	1.0733	1.0734	0.01	1.0118	1.4
	II	1.0739	1.0475	2.5	1.0143	5.7
Formic Acid	I	0.9790	0.9172	6.5	0.8963	8.8
	II	0.8915	0.9190	6.3	0.9379	5.1
Acetic Acid	I	1.1016	1.0575	4.1	0.9710	4.5
	II	1.0848	1.1229	3.5	1.0179	6.4
Butyric Acid	I	1.0824	1.0989	1.5	1.1844	9.0
	II	1.1869	1.1783	0.7	1.1238	5.5
Valeric Acid	I	1.0289	1.0231	0.56	0.9978	3.1
	II	1.1053	1.1003	0.45	1.0418	5.9

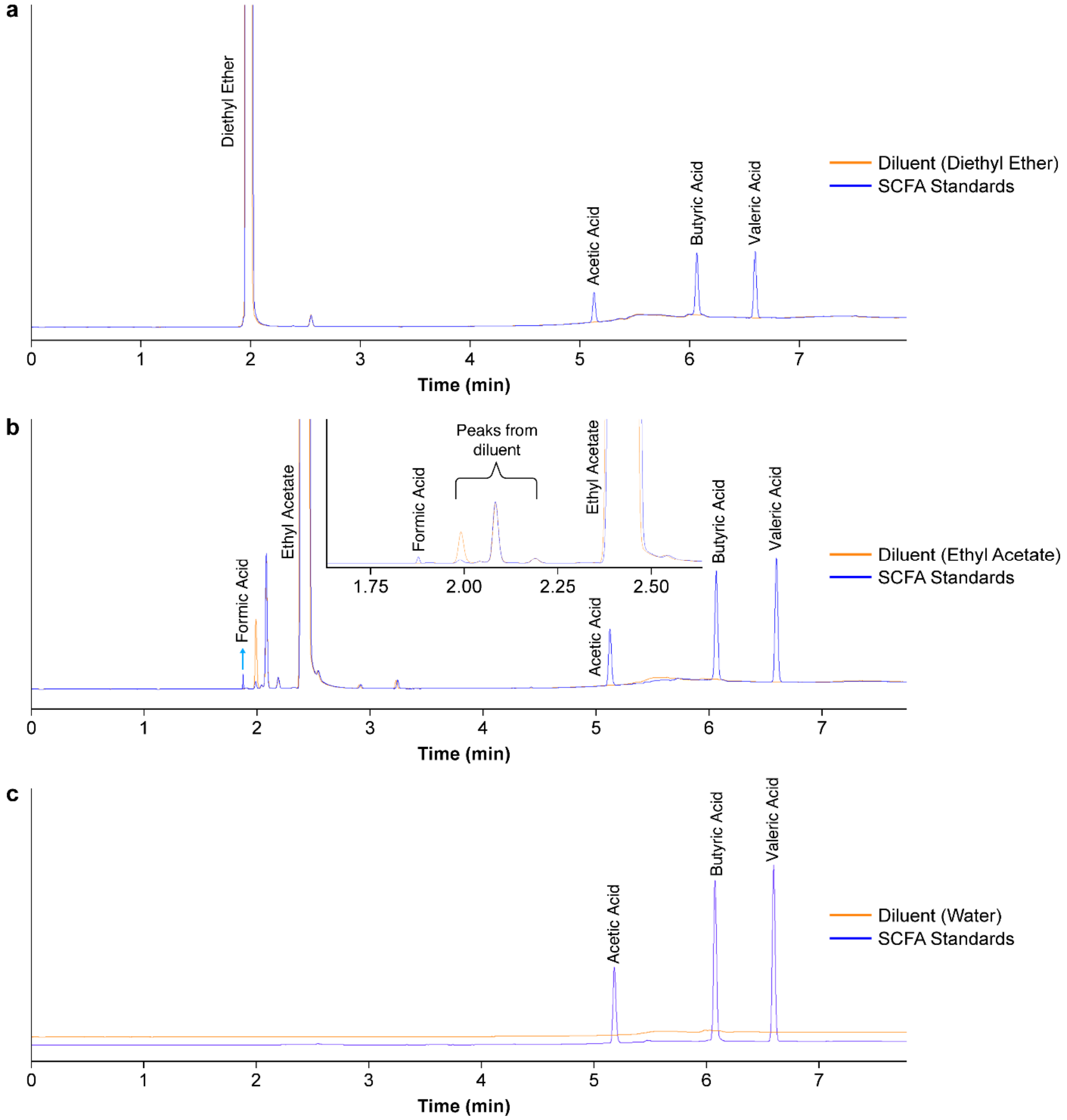

Figure S1. Typical GC-FID chromatograms of the SCFA analytes (formic acid, acetic acid, butyric acid, and valeric acid) diluted in **(a)** diethyl ether, **(b)** ethyl acetate, and **(c)** water. No formic acid peak was observed with diluents diethyl ether and water, and formic acid sensitivity was greatly reduced with diluent ethyl acetate. Several impurity peaks were also observed in the ethyl acetate diluent.

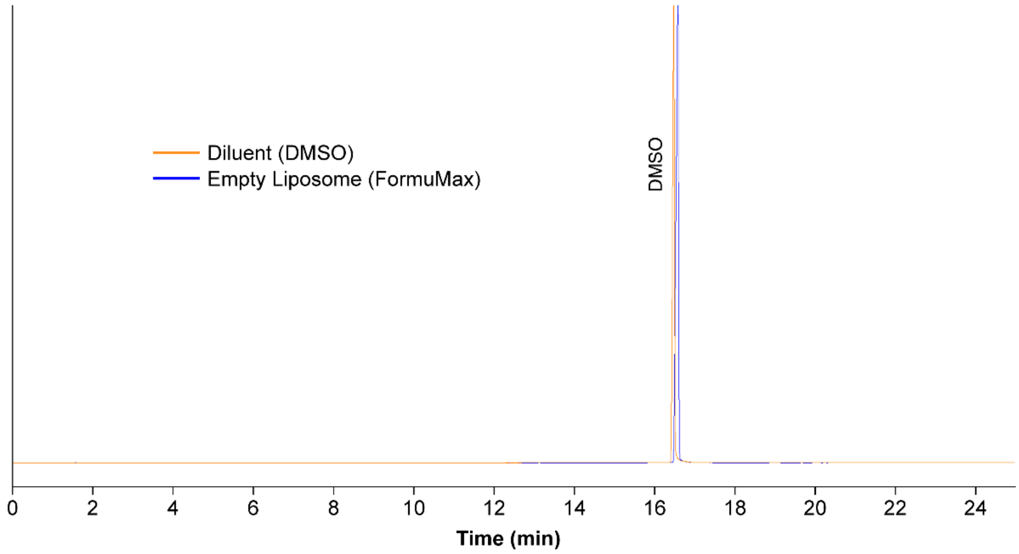

Figure S2. Typical GC-FID chromatograms of the DMSO diluent and FormuMax empty liposome diluted in DMSO. Both chromatograms show a single peak corresponding to the DMSO elution time. No additional peaks were observed, which would interfere with detection and quantitation of the other tested RS.

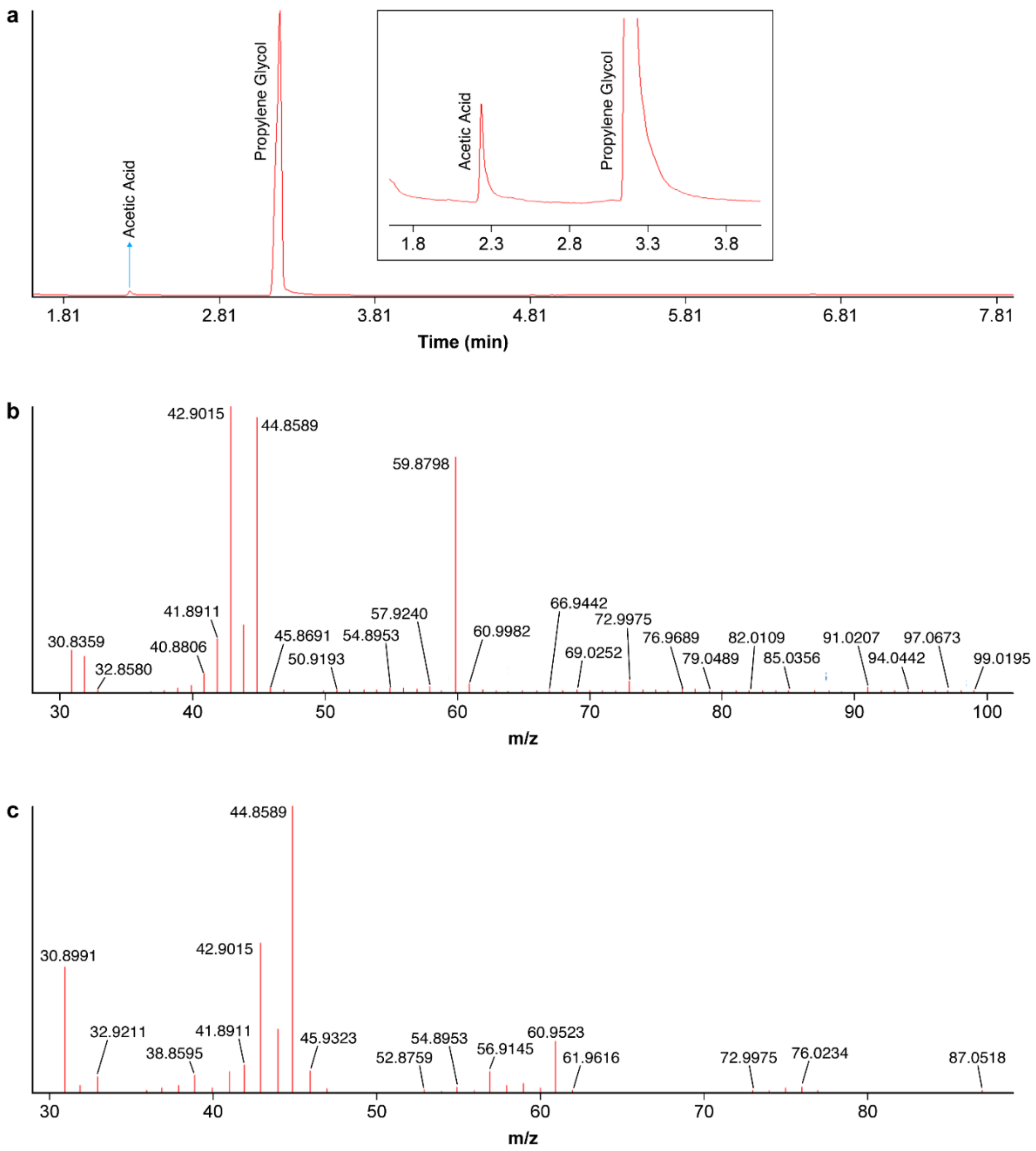

Figure S3. (a) Total ion chromatogram of the CLP formulation, showing the presence of acetic acid and propylene glycol. The inset depicts a zoomed chromatogram to enhance the acetic acid peak. **(b)** Molecular ion spectrum of the acetic acid peak showing an m/z of 59.88, including two major molecular ions—CH_3-CO^+ (m/z 42.90) and -$COOH^+$ (m/z 44.86). **(c)** Molecular ion spectrum of the propylene glycol peak showing an m/z of 76.02, including other major molecular ion fragments such as –CH_2OH^+ (m/z 30.90), CH_3-CO^+ (m/z 42.90), CH_3-CH_2OH^+ (m/z 44.86), CH_3-CH_2OH-CH_2^+ (m/z 60.95), and –CH_2OH-CH_2OH^+ (m/z 61.96).

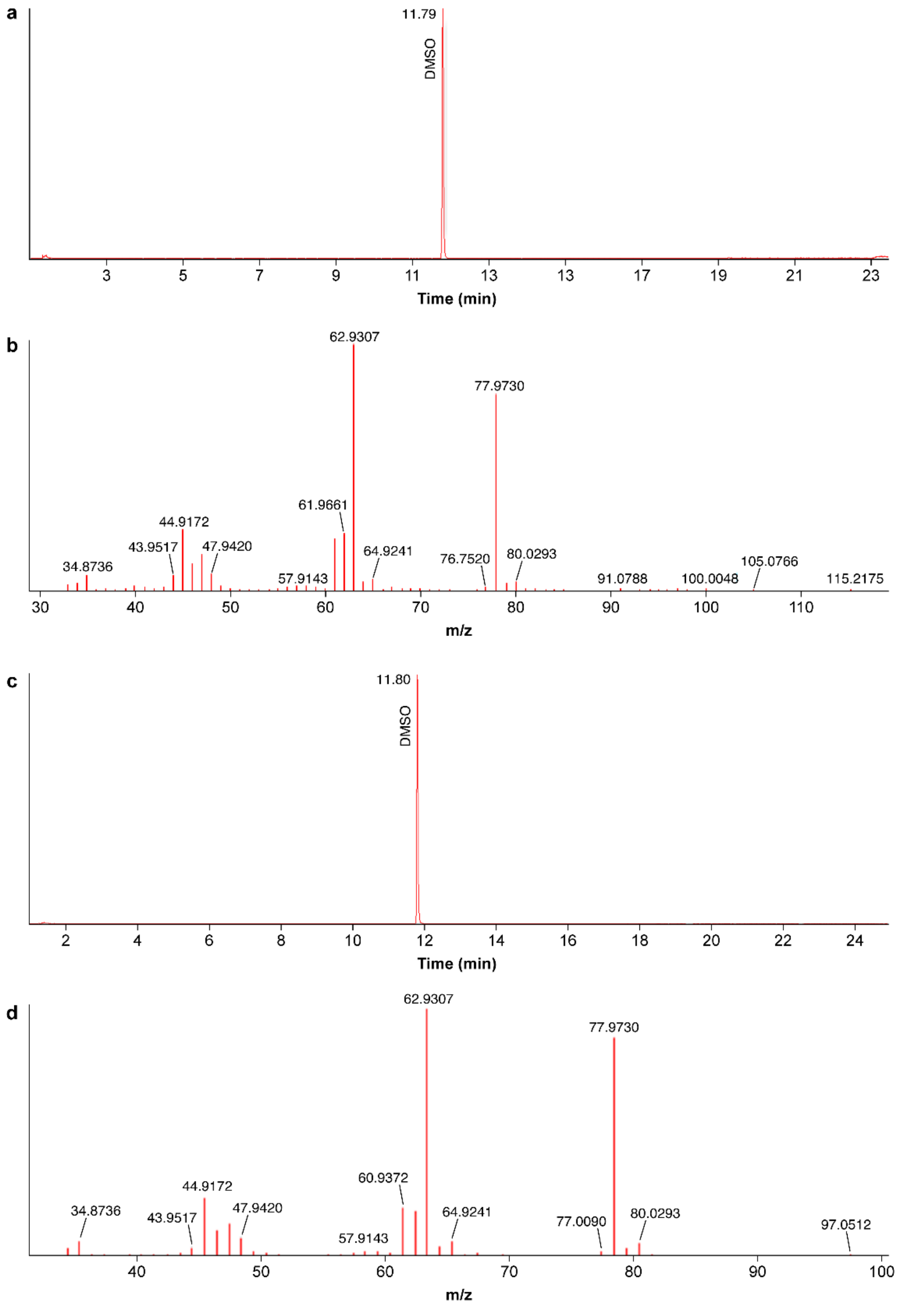

Figure S4. (a) Total ion chromatogram and **(b)** molecular ion spectra of the DMSO standard at 88 ppm, showing an m/z of 77.97 and the major fragment CH_3SO^+ (m/z 62.93). **(c)** Total ion chromatogram and **(d)** molecular ion spectra of the DM nanoconjugate, showing an m/z of 77.97 and the major fragment CH_3SO^+ (m/z 62.93).
